# Supplementary material for: The Novel Class IIa Selective Histone Deacetylase Inhibitor YAK540 Is Synergistic with Bortezomib in Leukemia Cell Lines
Source: Int J Mol Sci. 2022 Nov 2;23(21):13398. doi: 10.3390/ijms232113398 (PMC9656955; doi:10.3390/ijms232113398)
Supplement: Supplementary file 1 [file ijms-23-13398-s001.zip › ijms-1972058-supplementary.pdf]

# The novel Class IIa Selective Histone Deacetylase Inhibitor YAK540 is synergistic with Bortezomib in Leukemia Cell Lines

## Supplemental Information

**Experimental data**

**General methods:** All chemicals and solvents were purchased from commercial suppliers (Sigma Aldrich, Alfa Aesar, Fluorochem, TCI, abcr and Acros Organics) and used without further purification. All anhydrous reactions were carried out in flame-dried Schlenk-flasks and under argon atmosphere. Dry solvents were used directly from Seal® bottles from Acros Organics. Analytic Thin Layer Chromatography (TLC) was carried out with Macherey Nagel precoated silica gel plates (ALUGRAM® Xtra SIL G/$UV_{254}$). Detection was achieved with ultraviolet irradiation (254 nm) and/or staining with potassium permanganate solution (9 g $KMnO_4$, 60 g $K_2CO_3$, 15 mL of a 5% aqueous NaOH-solution, and 900 mL demineralised water). Flash column chromatography was performed with CombiFlashRf200 (TeleDyneIsco) with the solvent mixtures specified in the corresponding procedure.

***Physical data:*** Proton ($^1$H) and carbon ($^{13}$C) NMR spectra were recorded on Bruker Avance III – 300, Bruker Avance DRX – 500 or Bruker Avance III – 600. Spectra were referenced to the residual non-deuterated solvent signal ($^1$H-NMR: DMSO-*d*$_6$ (2.50 ppm), $^{13}$C-NMR: DMSO-d$_6$ (39.52 ppm); $^1$H-NMR: $CDCl_3$ (7.26 ppm), $^{13}$C-NMR: $CDCl_3$ (77.16 ppm)). Chemical shifts are quoted in parts per million (ppm). Signal patterns are indicated as: singlet (s), doublet (d), triplet (t), quartet (q), or multiplet (m). Coupling constants, *J*, are measured in Hz. Proton ($^1$H) and carbon ($^{13}$C) NMR spectra were recorded by the NMR-Divisions of the Department of Chemistry (Heinrich Heine University Duesseldorf). Electrospray Ionisation (ESI) mass spectra were carried out by the Mass spectrometry-Division of the Heinrich Heine University Duesseldorf, using Bruker Daltonics UHR-QTOF maXis 4G (Bruker Daltonics). APCI-MS was carried out using Advion expression$^L$ CMS spectrometer. Melting points (mp.) were determined using a Büchi M-565 melting point apparatus and are uncorrected. Analytical HPLC analysis were carried out on a Knauer HPLC system comprising an Azura P 6.1L pump, an Optimas 800 autosampler, a Fast Scanning Spectro-Photometer K-2600 and a Knauer Reversed Phase column (SN: FK36). UV absorption was detected at 254 nm. The solvent gradient table is shown below (Table S1). The purity of all final compounds was 95% or higher.

**Table S1.** The solvent gradient table for analytic HPLC analysis.

| Time / min | Water + 0.1% TFA | ACN + 0.1% TFA |
|---|---|---|
| Initial | 90 | 10 |
| 0.50 | 90 | 10 |
| 20.0 | 0 | 100 |
| 30.0 | 0 | 100 |
| 31.0 | 90 | 10 |
| 40.0 | 90 | 10 |

**Synthesis of 2-(2-(piperidin-1-yl)ethoxy)isoindoline-1,3-dione (2)**

Following the procedure of Asfaha et al.,[3] 1.00 eq of the respective halide derivative **1** and 1.20 eq *N*-hydroxyphtalimide (NHPI) were dissolved in acetonitrile (4 mL/mmol). After the addition of 2.00 eq triethylamine, the resulting solution was refluxed for 12 h. The solvent was evaporated under reduced pressure and the residue was diluted with EtOAc, washed with saturated $NaHCO_3$ solution (7x 50 mL),

dried over $Na_2SO_4$ and filtered. The crude product was purified by flash chromatography (n hexane/EtOAc/0.1% $NEt_3$) to afford **2.** Yellow solid, yield: 52%, mp: 91.1 °C. $^1$H NMR (300 MHz, DMSO-d6) δ = 1.18 – 1.31 (m, 6H), 2.27 (d, J = 4.6 Hz, 4H), 2.57 – 2.68 (m, 2H), 4.19 – 4.28 (m, 2H), 7.86 (s, 4H) ppm. $^{13}$C NMR (75 MHz, DMSO-d6) δ = 23.67, 25.23, 53.90, 57.03, 73.48, 123.01, 128.66, 134.56, 163.00 ppm.
HPLC analysis: Rt = 6.291 min, 92%. HRMS (ESI+) = calcd. for $C_{16}H_{23}N_2O_4$ $[M+H]^+$ = 307.1652, found: 307.1653.

**Synthesis of *O*-(2-(piperidin-1-yl)ethyl)hydroxylamine (3)**
To a solution of the respective NHPI-derivative **2** (1.00 eq) in $CH_2Cl_2$ (20 mL/mmol), 2.00 eq hydrazine monohydrate was added dropwise. The reaction mixture was stirred overnight at rt. The resulting precipitate was filtered, and the organic layer was washed with saturated $NaHCO_3$ solution (3x 10 mL). After drying over $Na_2SO_4$ and filtration, the solvent was removed *in vacuo* providing the hydroxylamine **3**. The hydroxylamine **3** was then used directly for the next step without any further purification.

**Synthesis of 4 -(N'-hydroxycarbamimidoyl)benzoic acid:** Following the procedure of Lobera et al.,[1] 1.00 eq 4-cyanobenzoic acid and 0.4 mol% 8-hydroxyquinoline were dissolved in EtOH (10 mL/mmol). To this reaction mixture 2.00 eq hydroxylamine hydrochloride in water (2.0 mL/mmol) and 1.60 eq potassium carbonate in water (1 mL/mmol) were added. The mixture was stirred at reflux for 6 h. The solvent was then removed under reduced pressure. The residue was diluted with water and the aqueous phase was acidified to pH = 3. The formed precipitate was then filtered, washed with water and acetone to furnish the corresponding amidoxime. White solid, yield: 86%. All spectroscopic data were in agreement with the literature. [1]

**Synthesis of 4-(5-(Trifluoromethyl)-1,2,4-oxadiazol-3-yl)benzoic acid (4):** A solution of corresponding amidoxime (1.00 eq) in anhydrous toluene (1 mmol/mL) was cooled to 0 °C and 3.00 eq trifluoroacetic anhydride was added dropwise. The reaction mixture was slowly allowed to warm to rt and then refluxed further for 8 h. The solvent was removed *in vacuo*, and the crude solid was diluted with ethyl acetate. The organic layer was washed with brine (3x25 mL), dried over $Na_2SO_4$, filtered and the solvent was then removed under reduced pressure. The crude product was purified by recrystallisation (ethyl acetate/ *n*-hexane) to yield the desired product **4**. White solid, yield: 60%, mp: 251 °C. $^1$H NMR (300 MHz, DMSO-d6) δ = 8.08 – 8.24 (m, 4H), 13.38 (s, 1H) ppm. $^{13}$C NMR (75 MHz, DMSO) δ = 115.65 (q), 127.56, 128.04, 130.20, 134.20, 165.47, 165.24 (q), 167.86 ppm. HPLC: $t_R$ = 12.483 min, AUC = 95.8%.

**Synthesis of YAK 540**
For the synthesis of **YAK 540**, 1.00 eq of the corresponding carboxylic acid **4** was dissolved in dry DMF (0.1 mmol/mL) and 1.00 eq HATU and 2.00 eq DIPEA were added. After stirring for 15 min at rt, the respective hydroxylamine **3** (1.00 eq) was then added and the resulting mixture was stirred for further 16h at rt. The solvent was removed in vacuo and the residue was diluted with EtOAc. The organic layer was washed with saturated $NaHCO_3$ (3x 50 mL), brine (1x 50 mL), dried over $Na_2SO_4$ and filtered. After removing the solvent, the crude product was purified as stated. The crude product was purified by flash chromatography ($CH_2Cl_2$/30% MeOH in $CH_2Cl_2$/ 0.1% $NEt_3$) to furnish **YAK 540**. Yellowish solid. yield: 54%, mp.: 131 °C.
$^1$H NMR (300 MHz, DMSO-$d_6$) δ = 1.41 (q, *J* = 6.0 Hz, 2H), 1.55 (p, *J* = 5.5 Hz, 4H), 2.57 (t, *J* = 5.3 Hz, 4H), 2.71 (t, *J* = 5.5 Hz, 2H), 4.06 (t, *J* = 5.5 Hz, 2H), 7.95 – 8.01 (m, 2H), 8.12 – 8.20 (m, 2H) ppm. $^{13}$C NMR (75 MHz, DMSO-$d_6$) δ = 23.39, 24.95, 53.82, 56.02, 72.27, 115.70 (d, *J* = 273.2 Hz), 126.91, 127.47, 128.18, 135.88, 163.10, 165.18 (d, *J* = 43.7 Hz), 167.86 ppm. HPLC analysis: $R_t$ = 10.321 min, 96.2%. HRMS (ESI+) = calcd. for $C_{17}H_{20}F_3N_4O_3$ $[M+H]^+$ = 385.1482, found: 385.1482.

**Concentration-response-curves HEK293**

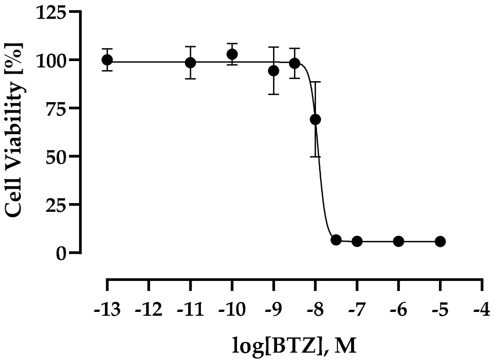


(**a**)

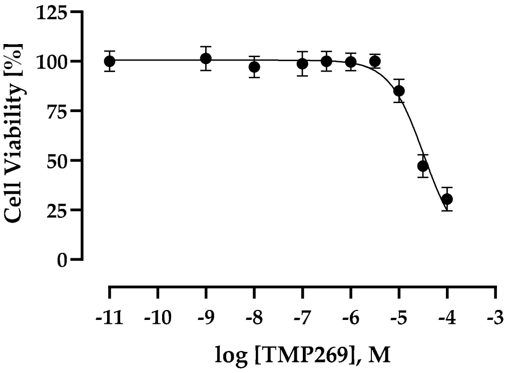


(**b**)

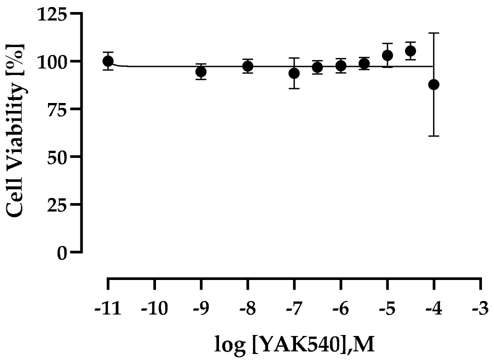


(c)

**Figure S1.** (a)–(c) Concentration-response-curves of HEK293. The data shown are from an n ≥ 8 from at least 3 independent experiments.

**Western Blot:**

**Table S2.** Listing of primary antibodies for the western blot

| **Protein of interest** | **expected size (kDa)** | **Primary antibody (company, Catalog nr.)** |
|---|---|---|
| Survivin | 16 | R&D systems, AF886 |
| APAF-1 | 130-140 | R&D systems, MAB868 |
| PARP | 116 | R&D systems, AF-600-NA |
| HDAC 4 | 140 | R&D systems, AF6205 |
| Beta Aktin | 45 | Cell Signaling Technologie, 13E5 |
| BAD | 22 | R&D systems, AF819 |
| p21 | 18 | Cell Signaling Technology #2947 |
| Phospho-Histone H2AX | 15 | R&D systems, AF2288 |
| Bcl-2 | 25 | R&D systems, AF810 |

**Uncropped western blots:**

HL-60 General Order of samples (24 h, 48 h incubation):

1. control
2. 10 nM bortezomib
3. 10 μM YAK540
4. 10 nM bortezomib + 10 μM YAK540
5. 5 μM TMP269
6. 10 nM bortezomib + 5 μM TMP269

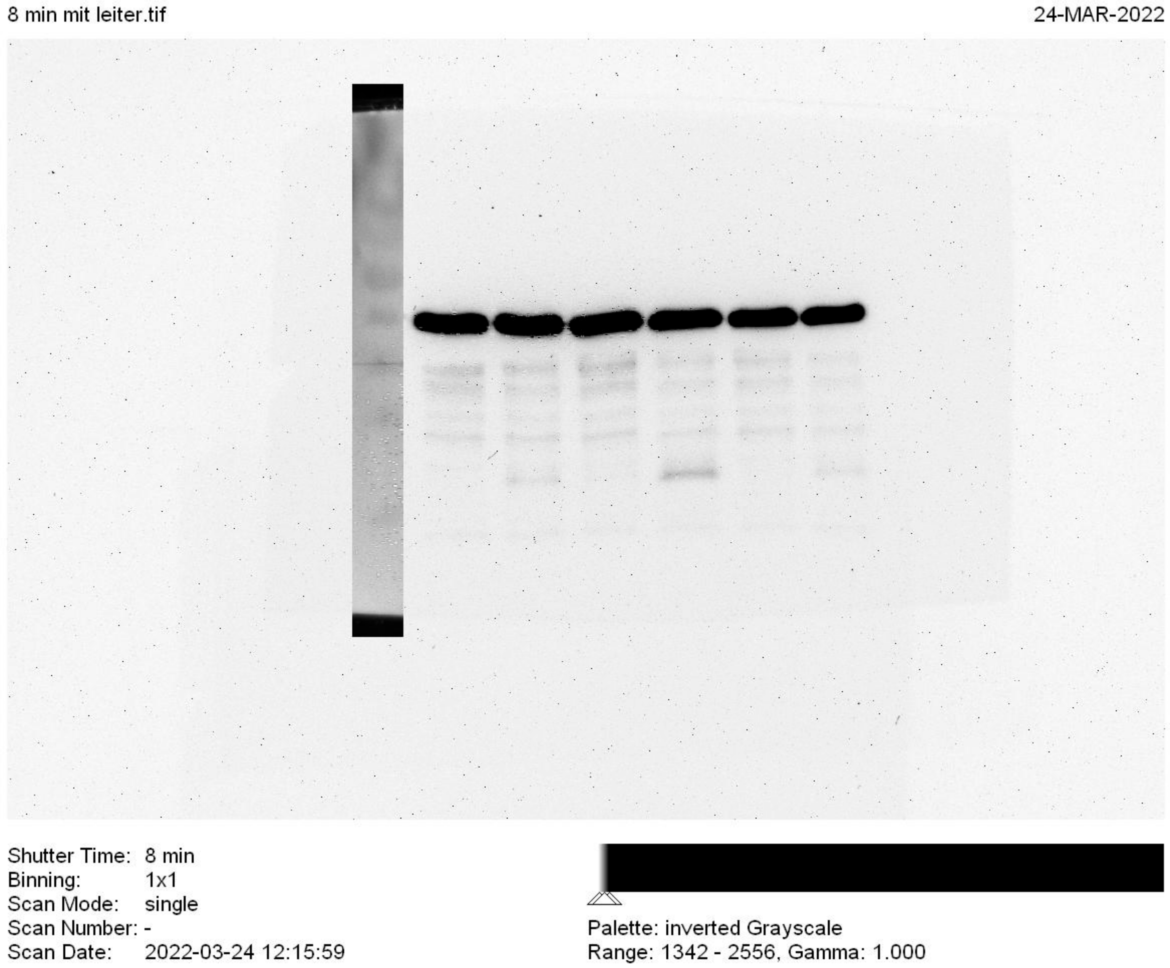


**Figure S2.** Uncropped western blot, HL-60, blot shows β-Aktin and p21, 24 h incubation.

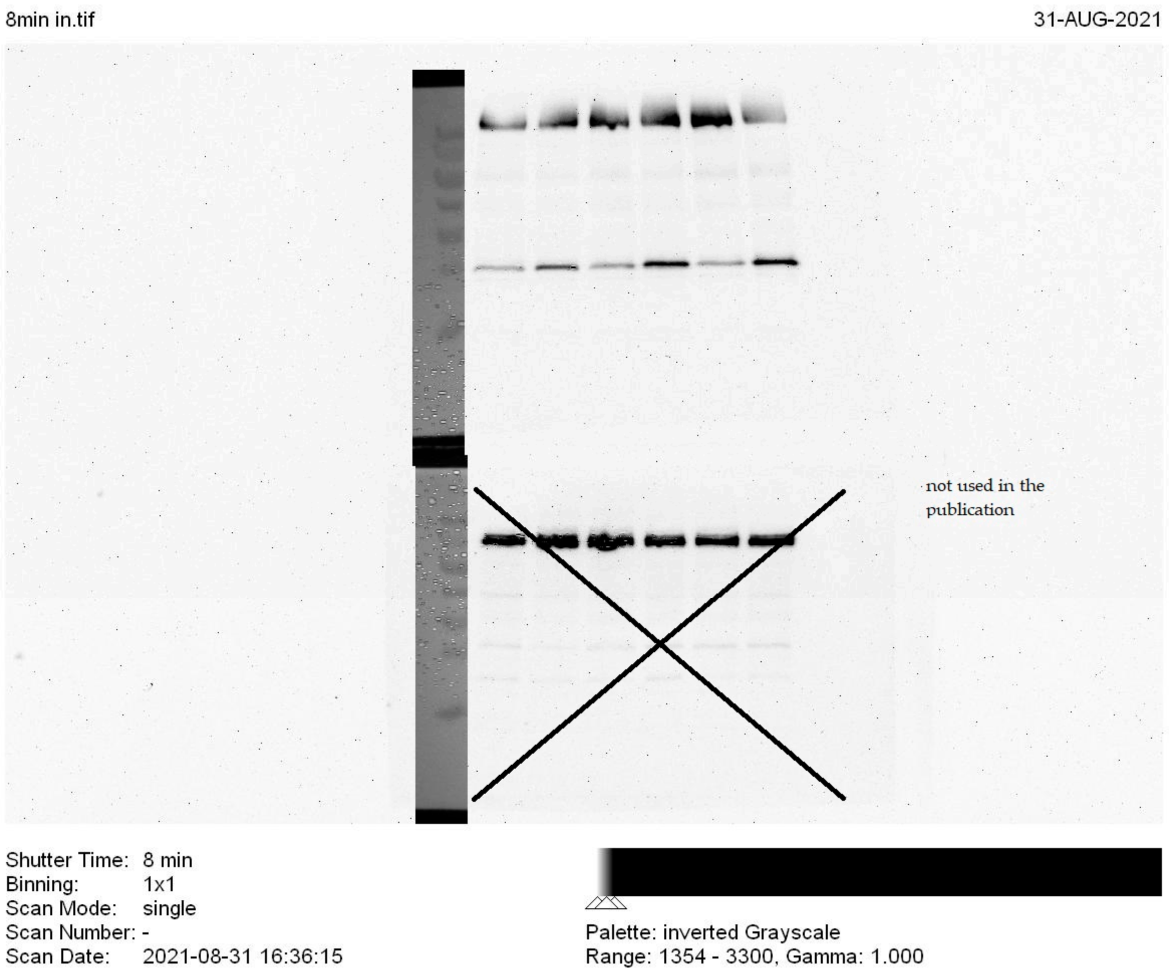


**Figure S3.** Uncropped western blot HL-60 blot at top shwos PARP, 24 h incubation.

single 31-AUG-2021

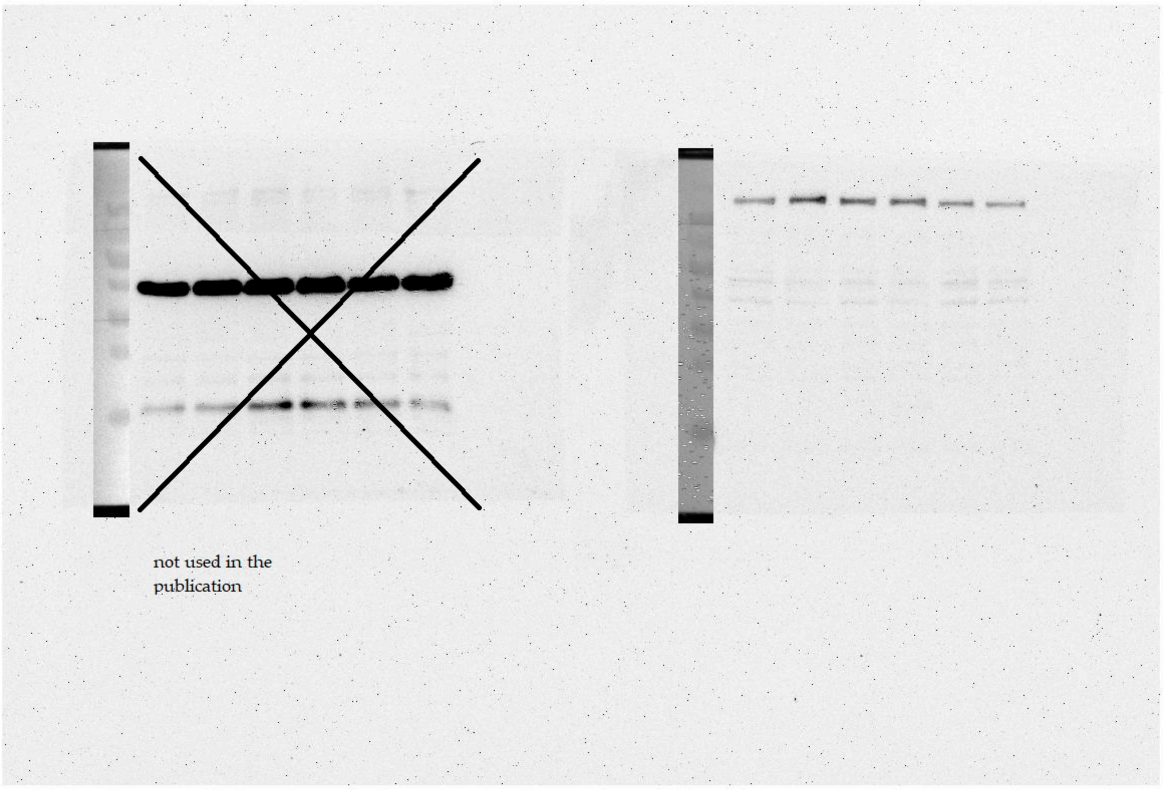


Shutter Time: 8 min
Binning: 1x1
Scan Mode: single
Scan Number: -
Scan Date: 2021-08-31 15:15:49

Palette: inverted Grayscale
Range: 1358 - 2269, Gamma: 1.000

**Figure S4.** Uncropped western blot, HL-60, right blot shows at top HDAC 4, 24 h incubation.

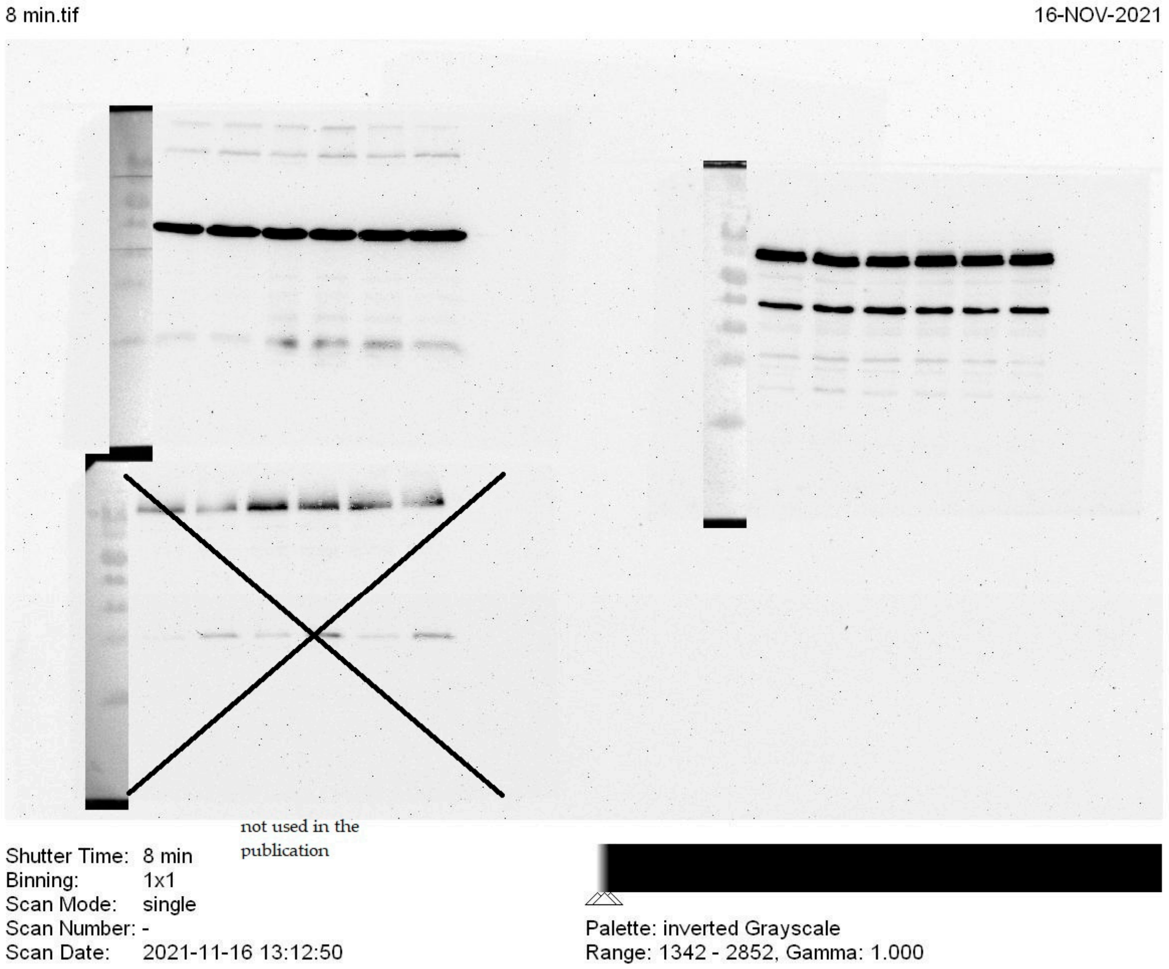


**Figure S5.** Uncropped western blot, HL-60, blot top left shows APAF-1 and Survivin, right blot illustrate Bad, 24 h incubation.

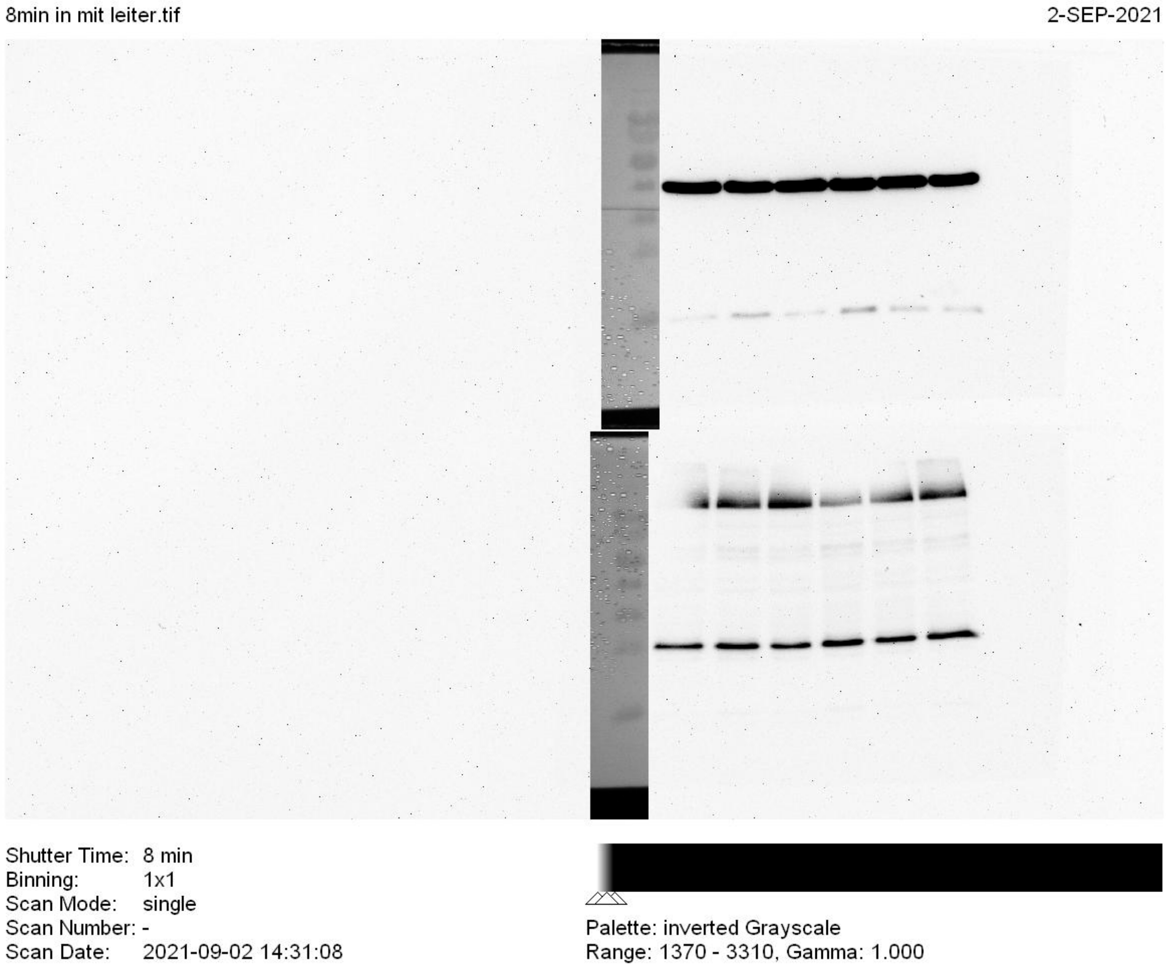


**Figure S6.** Uncropped western blot, HL-60, blot at top shows β-Actin and γ-H2AX, the blot below illustrate PARP, 48 h incubation.

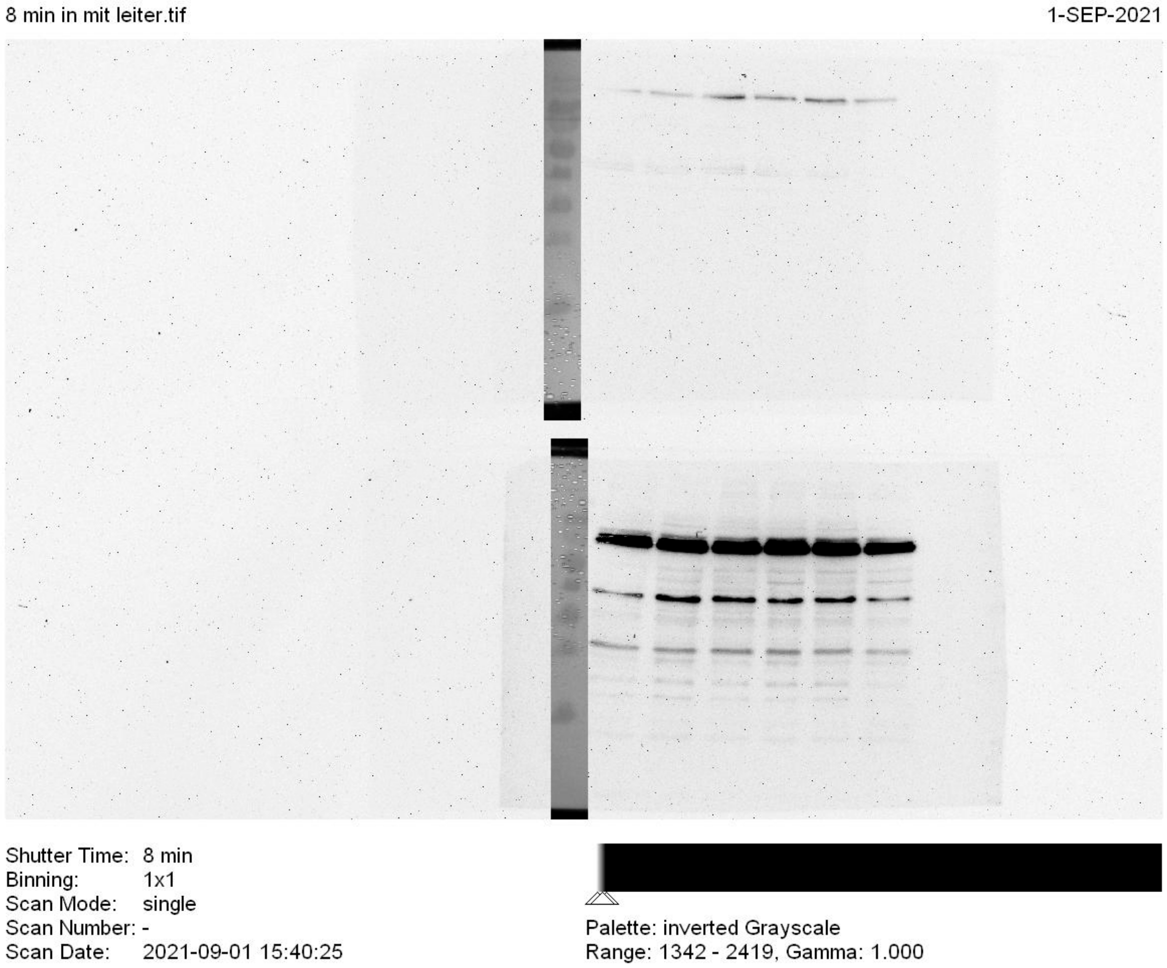


**Figure S7.** Uncropped western blot, HL-60, blot at top shows HDAC 4, blot below illustrate Bad, 48 h incubation.

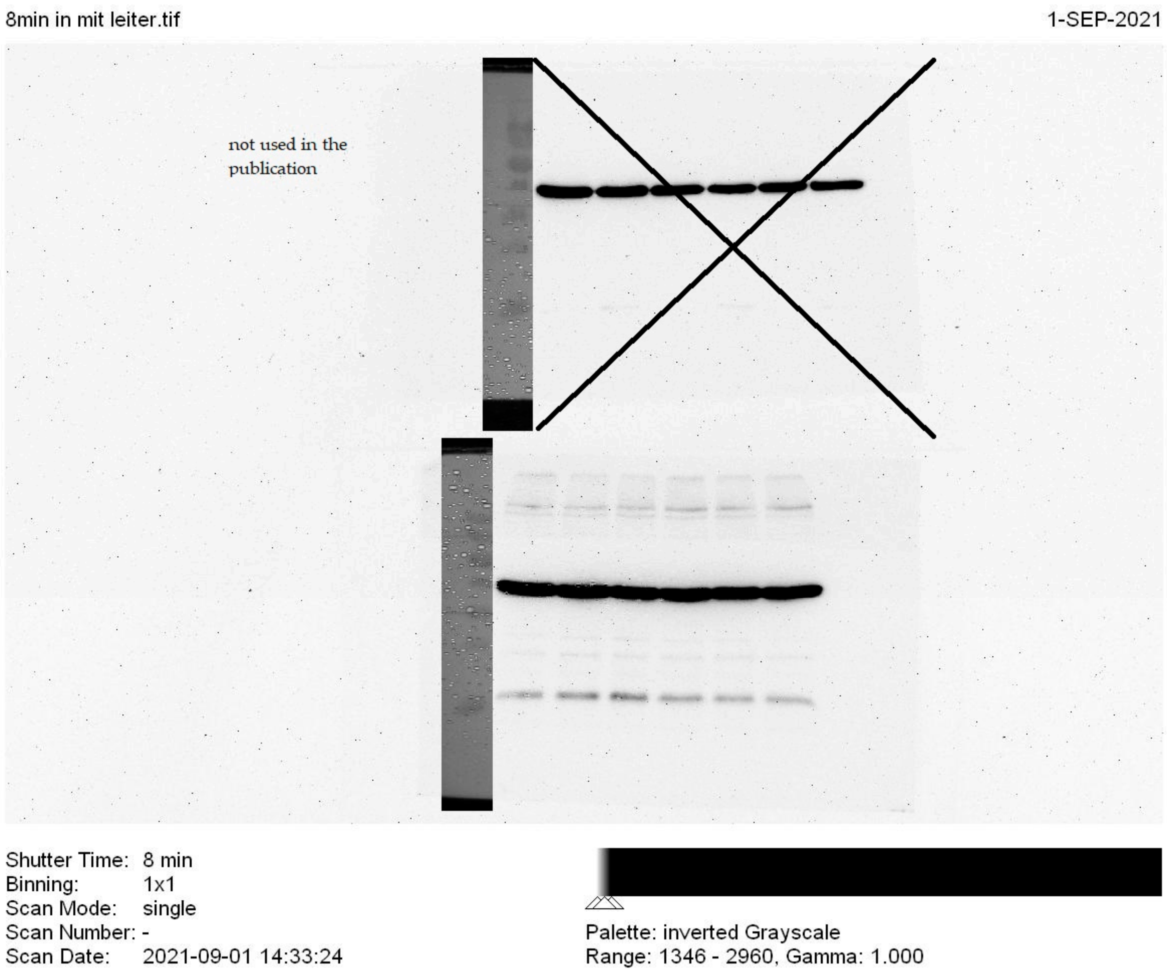


**Figure S8.** Uncropped western blot, HL-60 lower blot shows APAF-1 and Survivin, 48 h incubation.

K562 General Order of samples (24 h, 48 h incubation):

1. Control
2. 25 nM bortezomib
3. 10 µM YAK540
4. 10 µM YAK540 + 10 nM bortezomib
5. 5 µM TMP269
6. 25 nM bortezomib + 5 µM TMP269

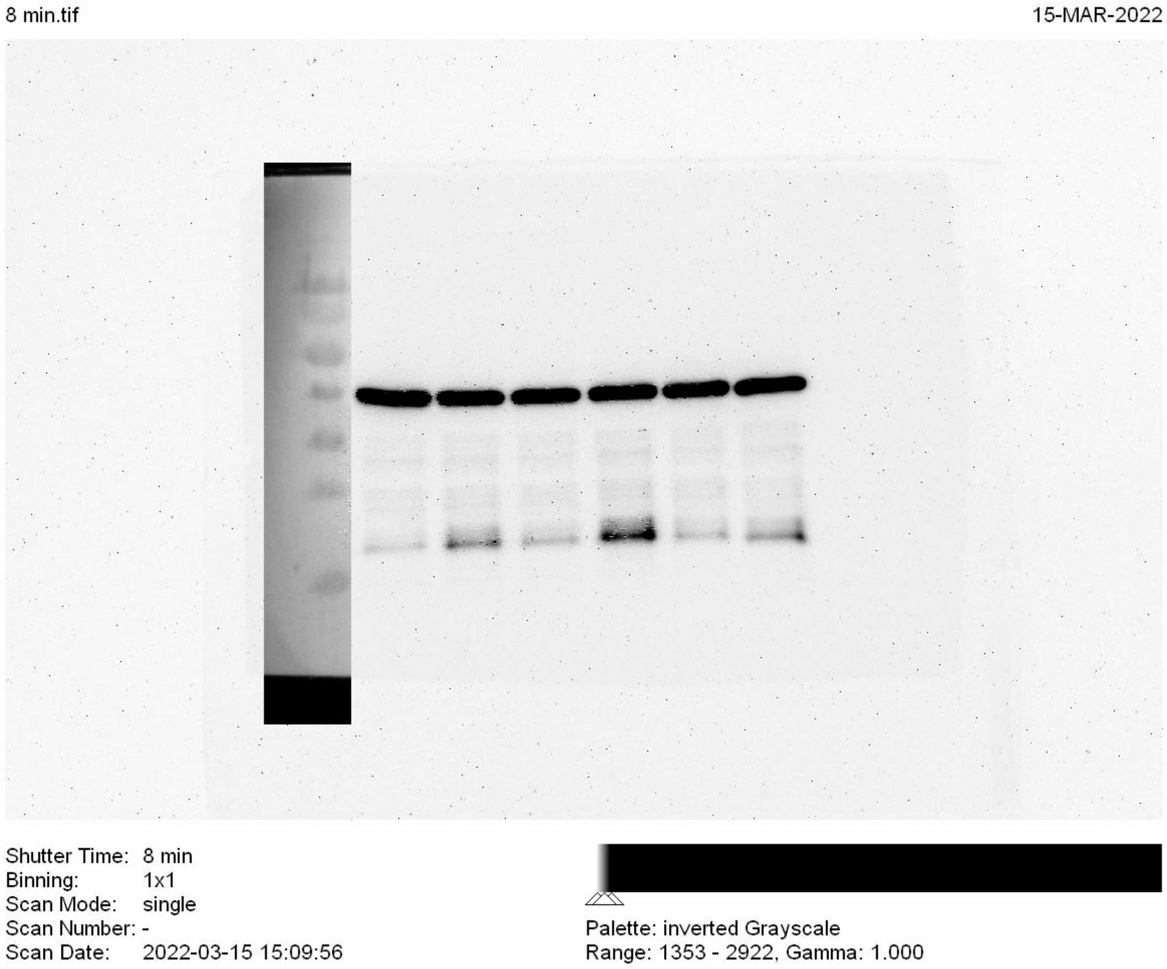


**Figure S9.** Uncropped western blot, K562, the blot shows β-Actin and p21, 24 h incubation.

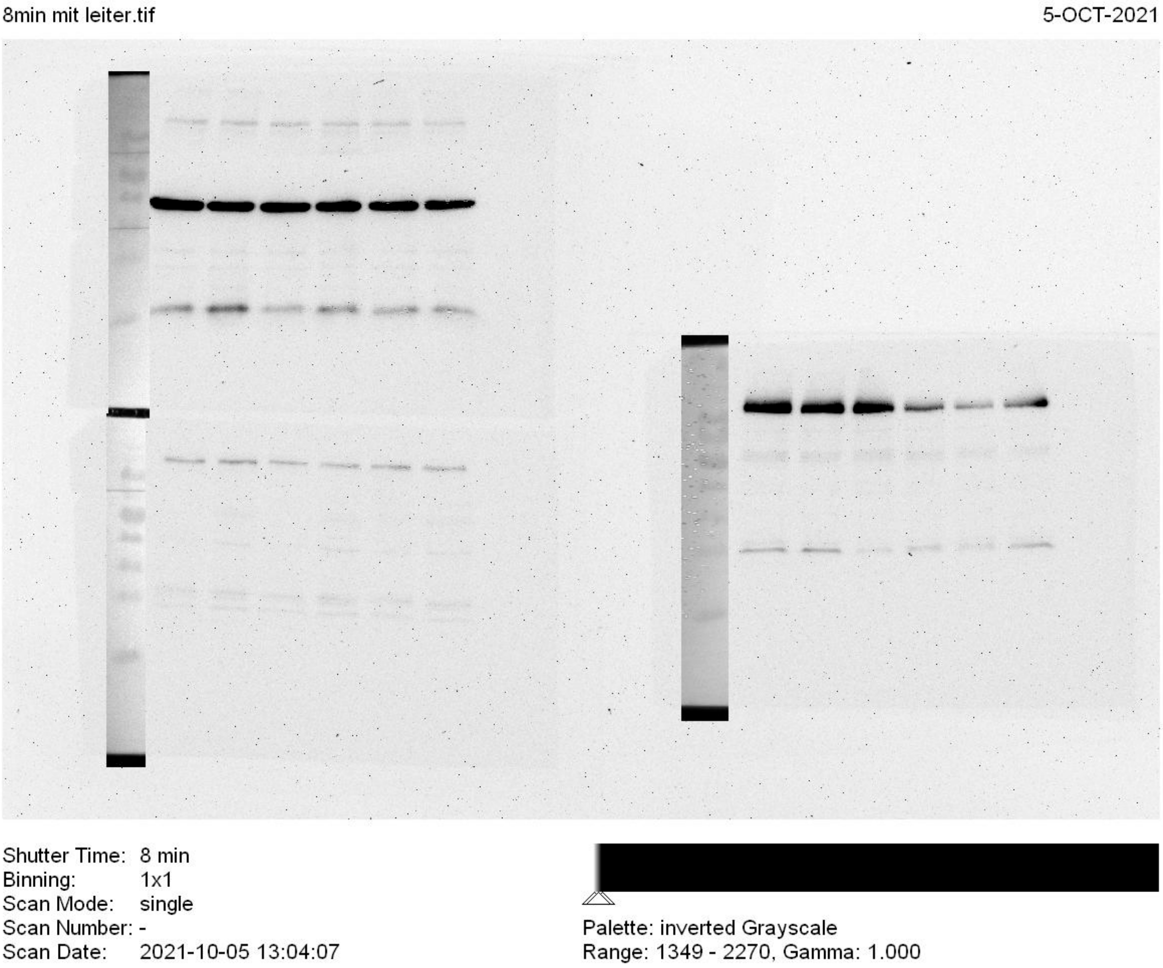


**Figure S10.** Uncropped western blot, K562 blot on the top left shows APAF-1, the blot below illustrate HDAC 4 and Bcl-2, the blot on the right side displays PARP, 24 h incubation.

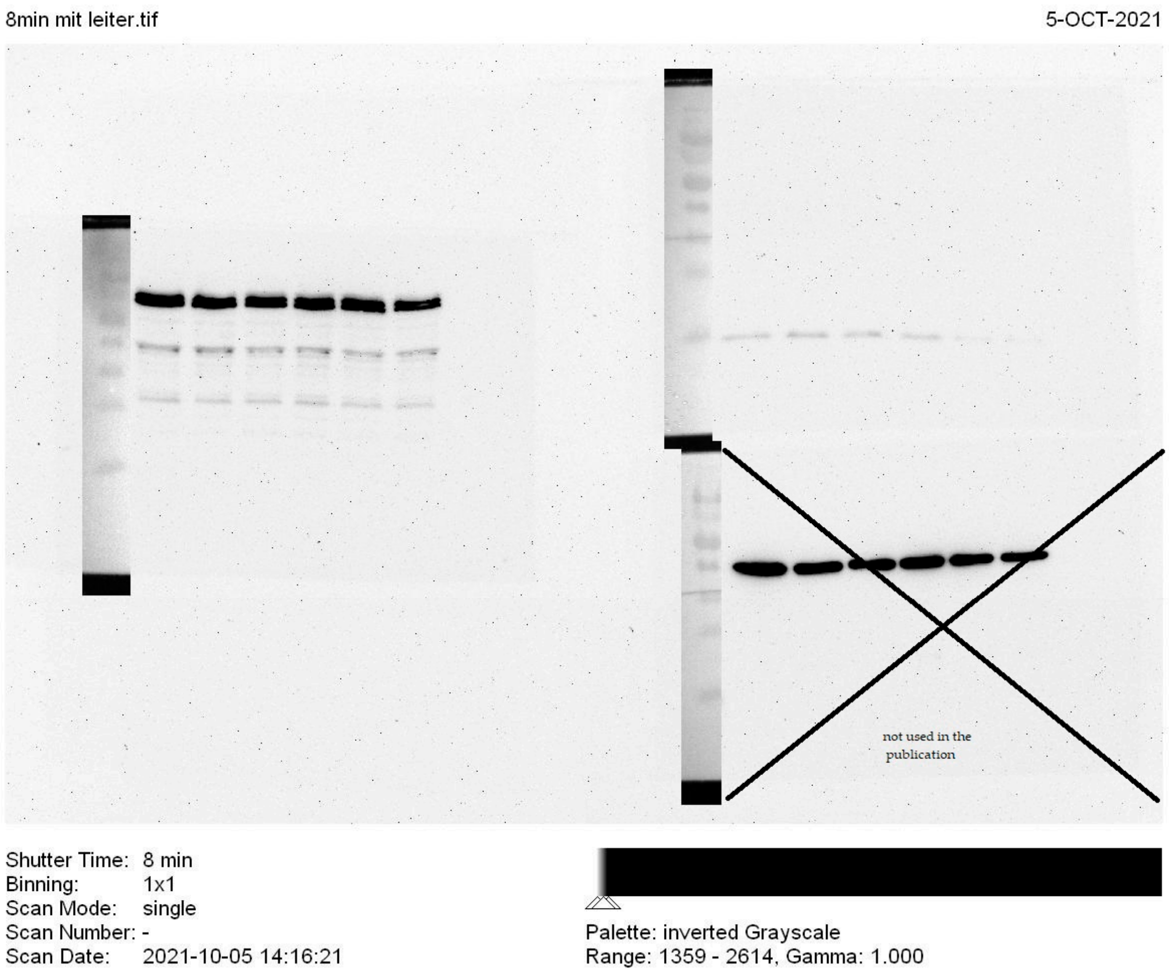


**Figure S11.** Uncropped western blot, K562, left blot shows Bad, the blot on top right illustrate γ-H2AX, 24 h incubation.

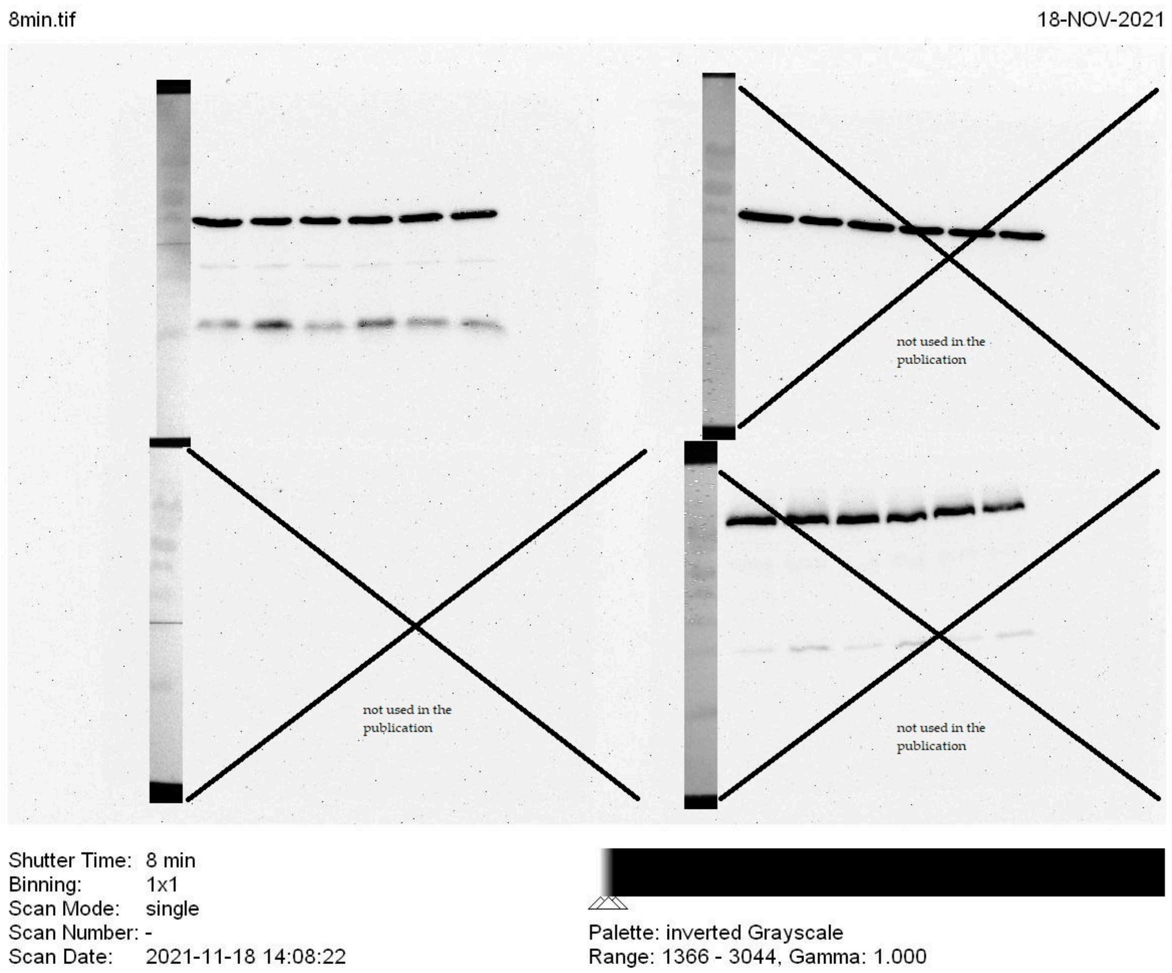


**Figure S12.** Uncropped western blot, K562, blot top left shows Survivin, 24 h incubation.

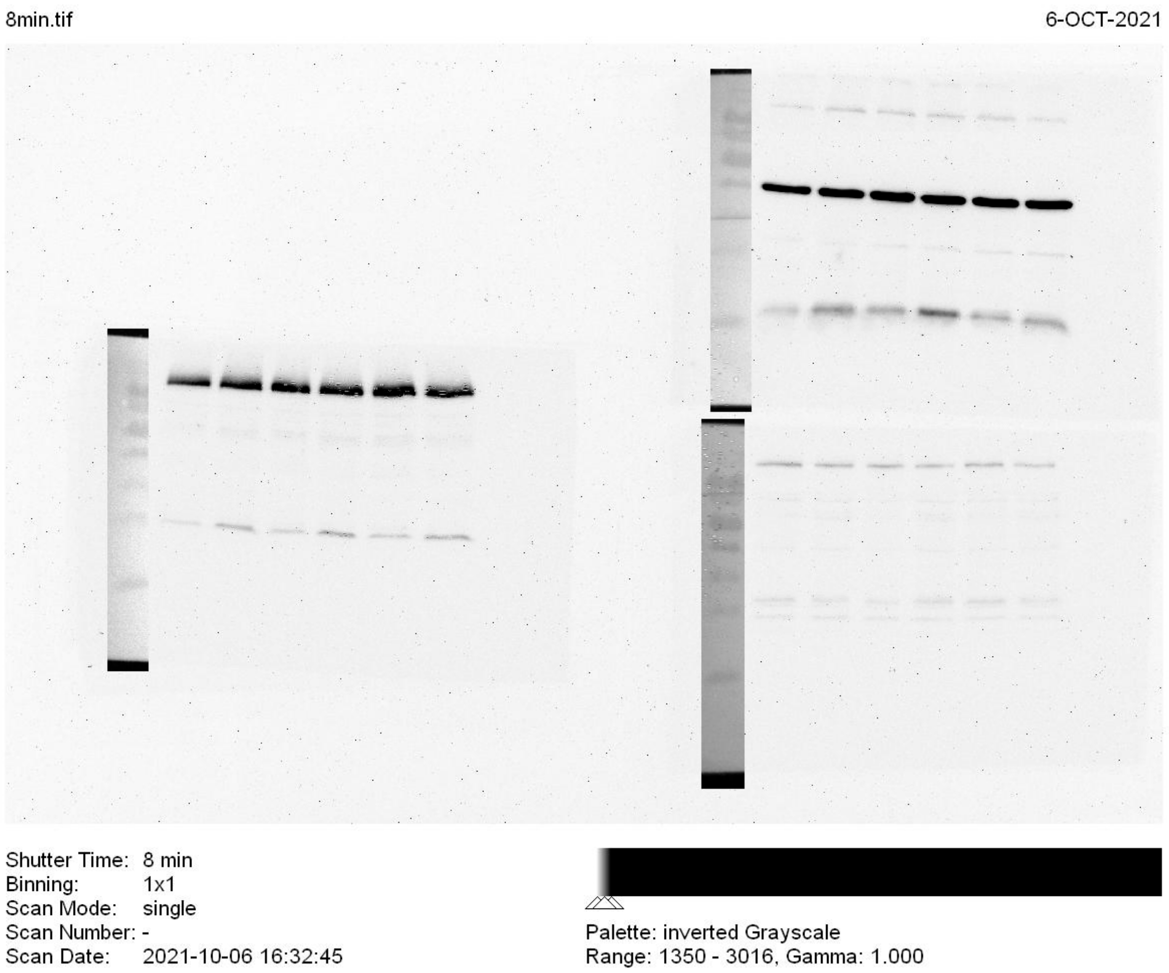


**Figure S13.** Uncropped western blot, K562, blot on the left side shows PARP, blot top right illustrate APAF-1 and β-Actin and Survivin, the blot below displays HDAC 4 and Bcl-2, 48 h incubation.

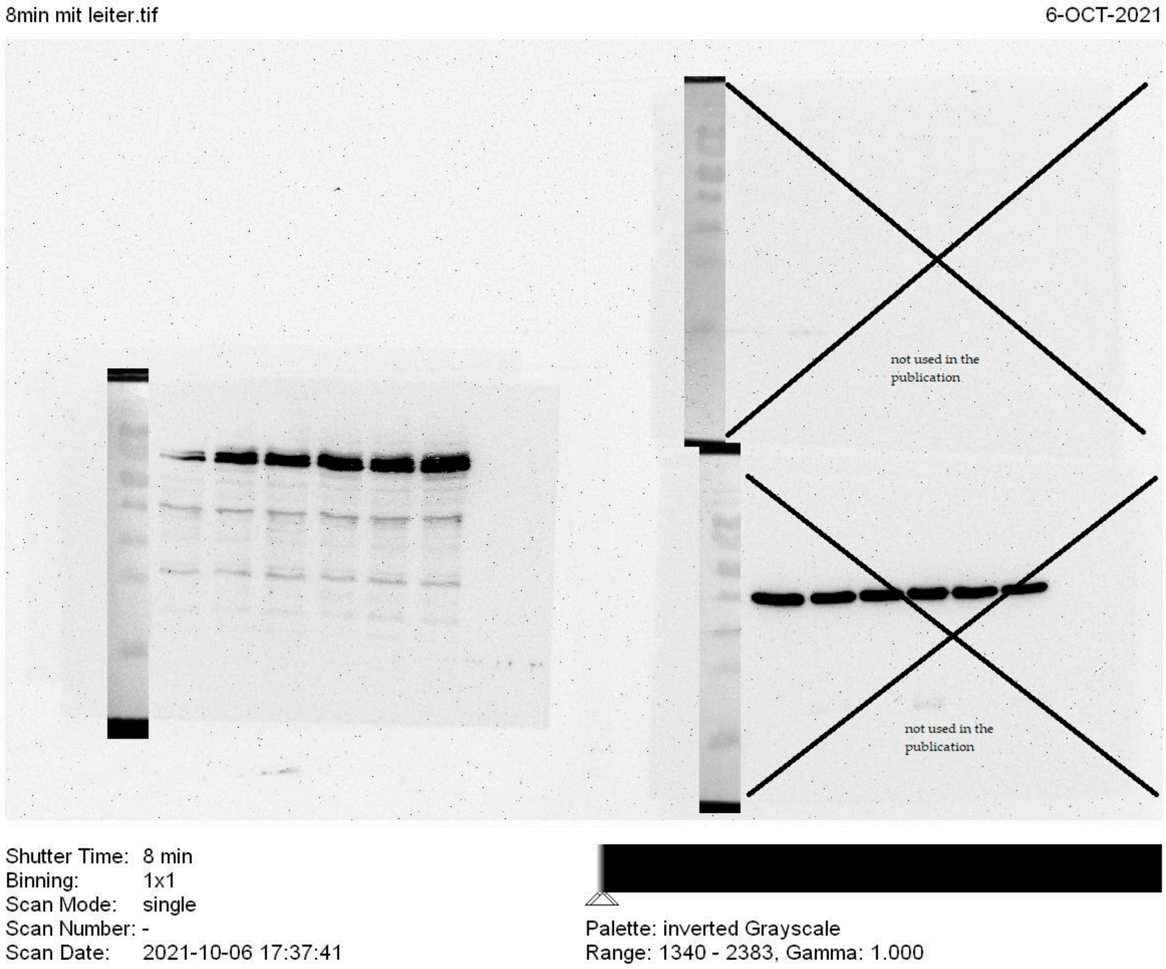


**Figure S14.** Uncropped western blot, K562, blot left side shows Bad, 48 h incubation.

MONO-MAC-6 General Order of samples (24 h, 48 h coincubation):

1. Control
2. 10 nM bortezomib
3. 10 µM Yak540
4. 10 nM bortezomib + 10 µM YAK540
5. 5 µM TMP269
6. 10 nM bortezomib + 5 µM TMP269

8 min mit leiter.tif 22-MAR-2022

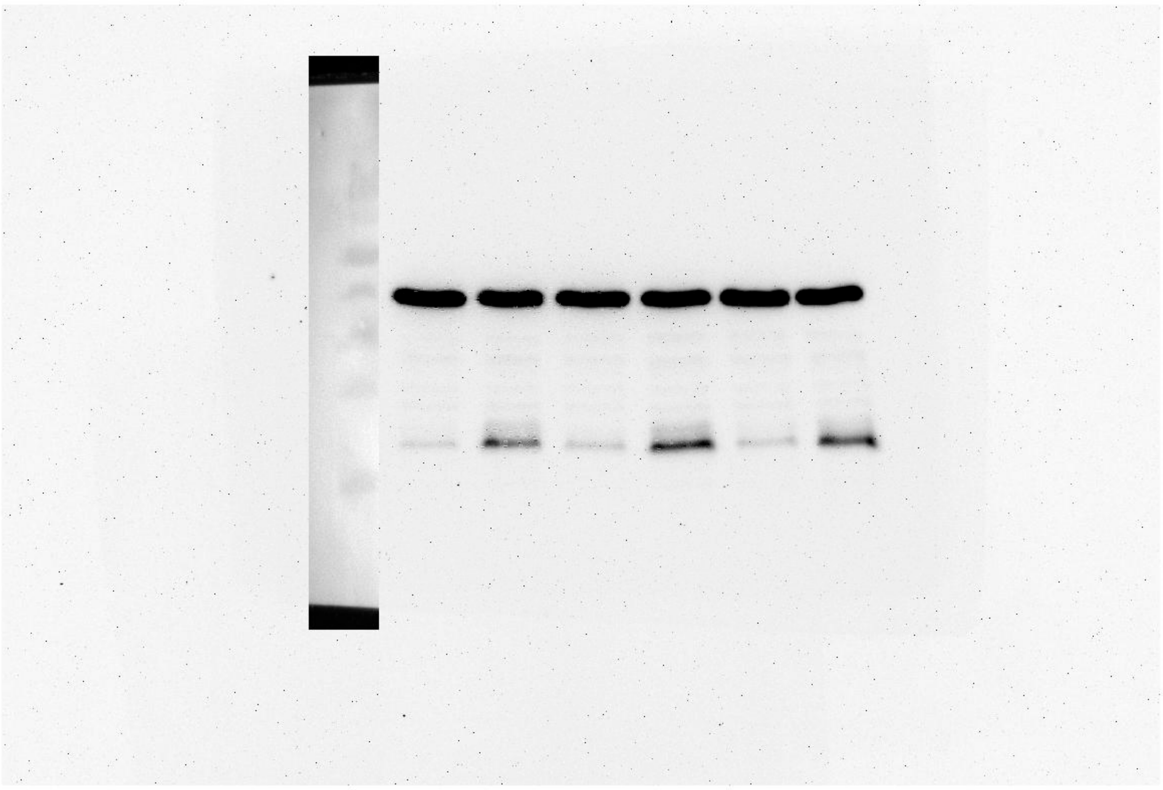

Shutter Time: 8 min
Binning: 1x1
Scan Mode: single
Scan Number: -
Scan Date: 2022-03-22 12:29:26

Palette: inverted Grayscale
Range: 1346 - 2714, Gamma: 1.000

**Figure S15.** Uncropped western blot, MONO-MAC-6, blot shows β-Actin and p21, 24 h incubation.

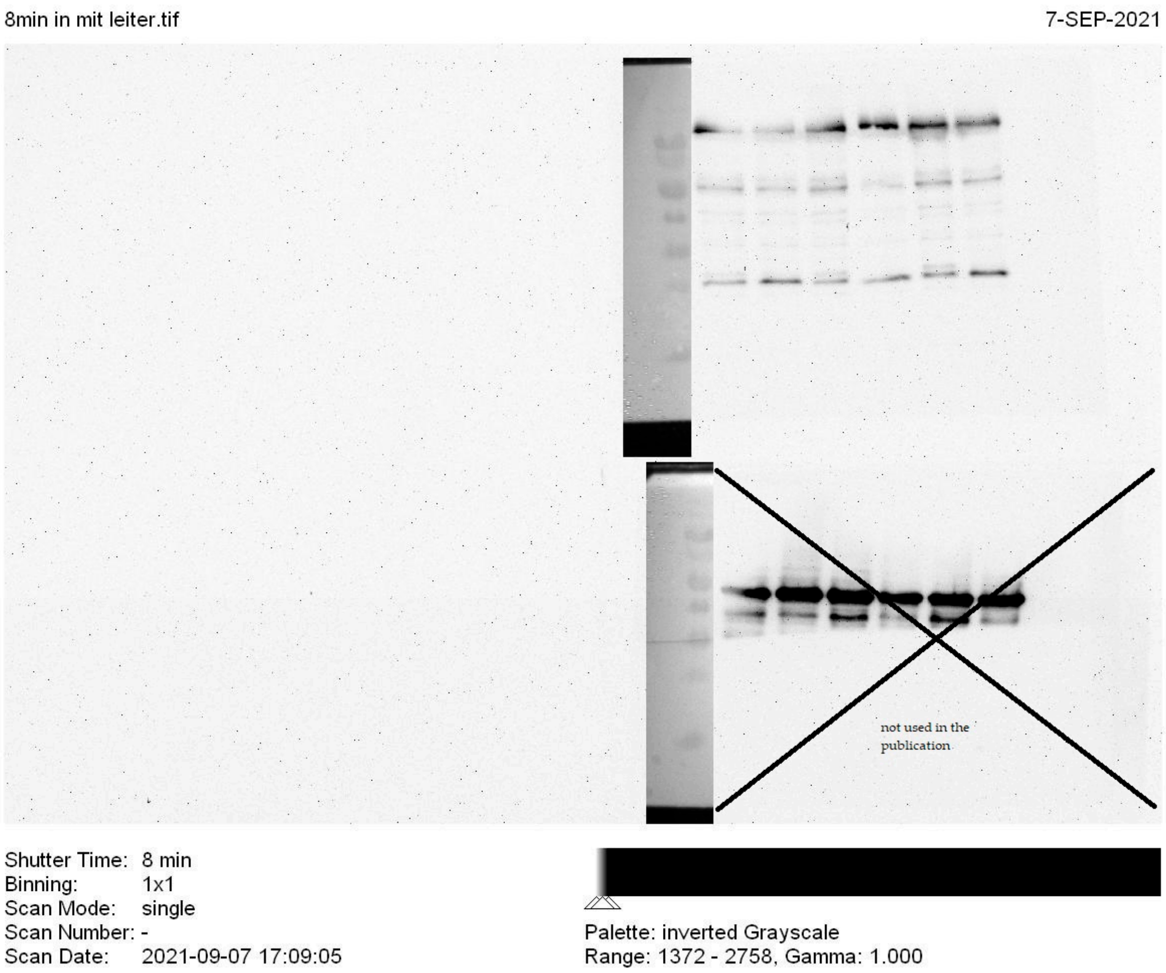


**Figure S16.** Uncropped western blot, MONO-MAC-6, blot at top shows PARP, 24 h incubation.

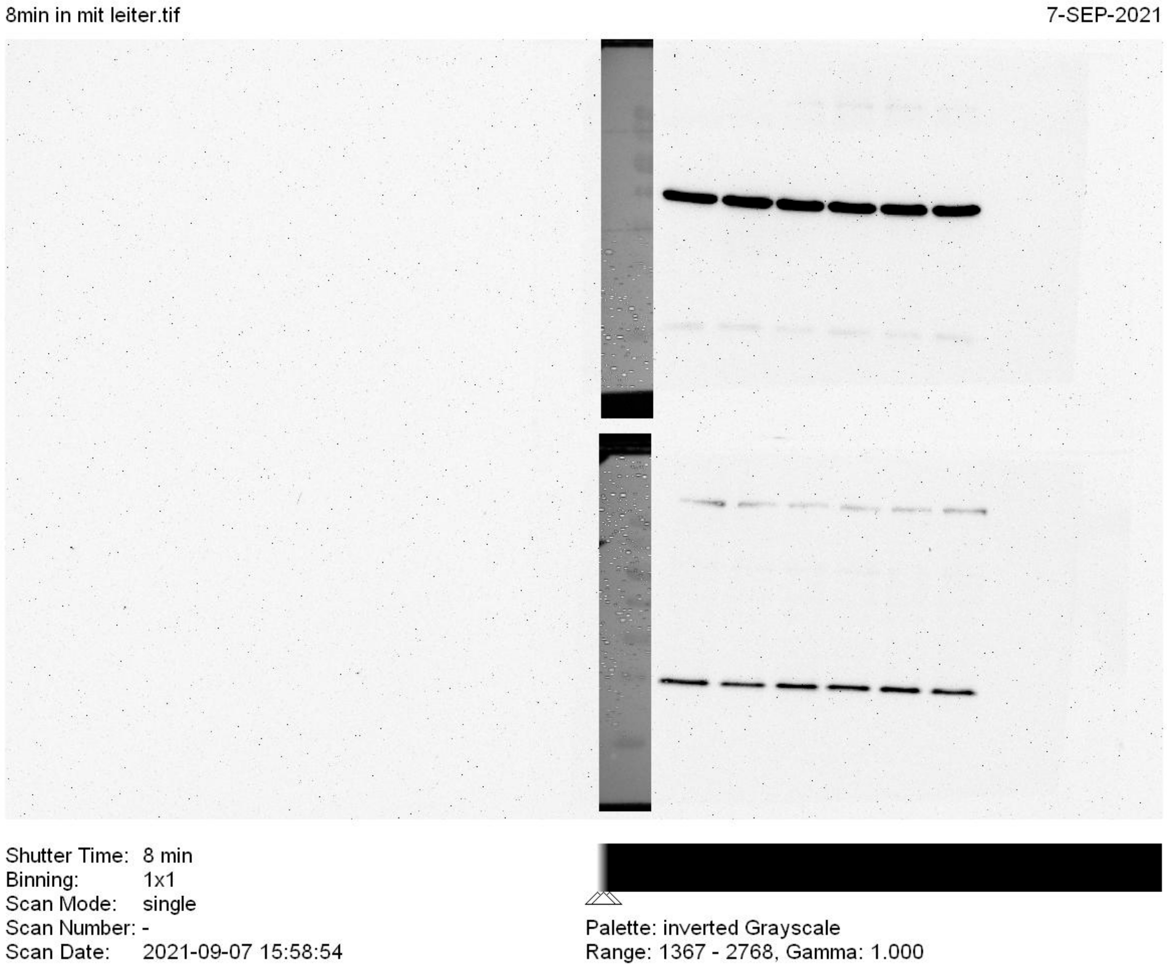


**Figure S17.** Uncropped western blot, MONO-MAC-6, blot at top shows Survivin, blot below illustrate HDAC 4 and Bcl-2, 24 h incubation.

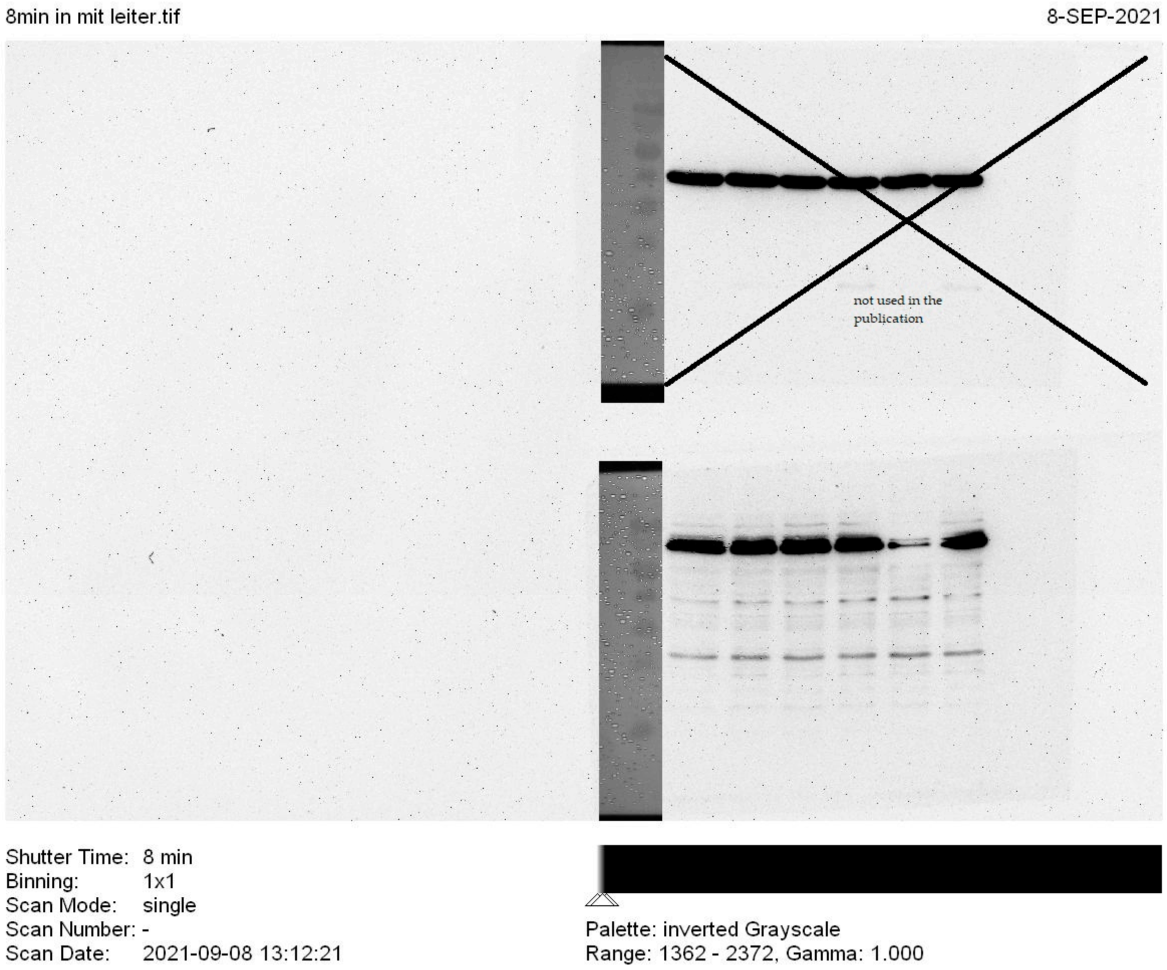


**Figure S18.** Uncropped western blot, MONO-MAC-6, lower blot shows Bad, 24 h incubation.

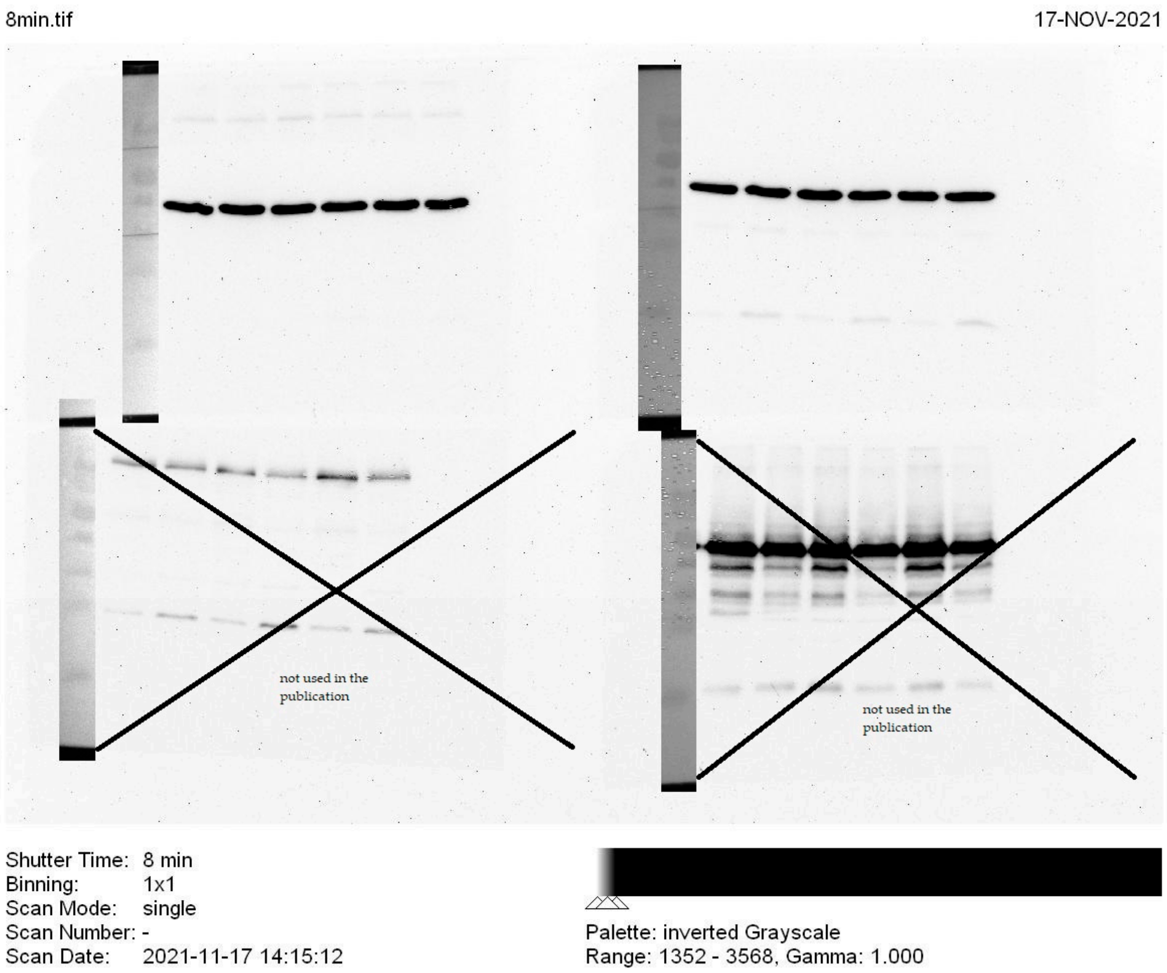


**Figure S19.** Uncropped western blot, MONO-MAC-6, blot top left shows APAF-1, 24 h icubation; blot top right illustrate β-Actin, 48 h incubation.

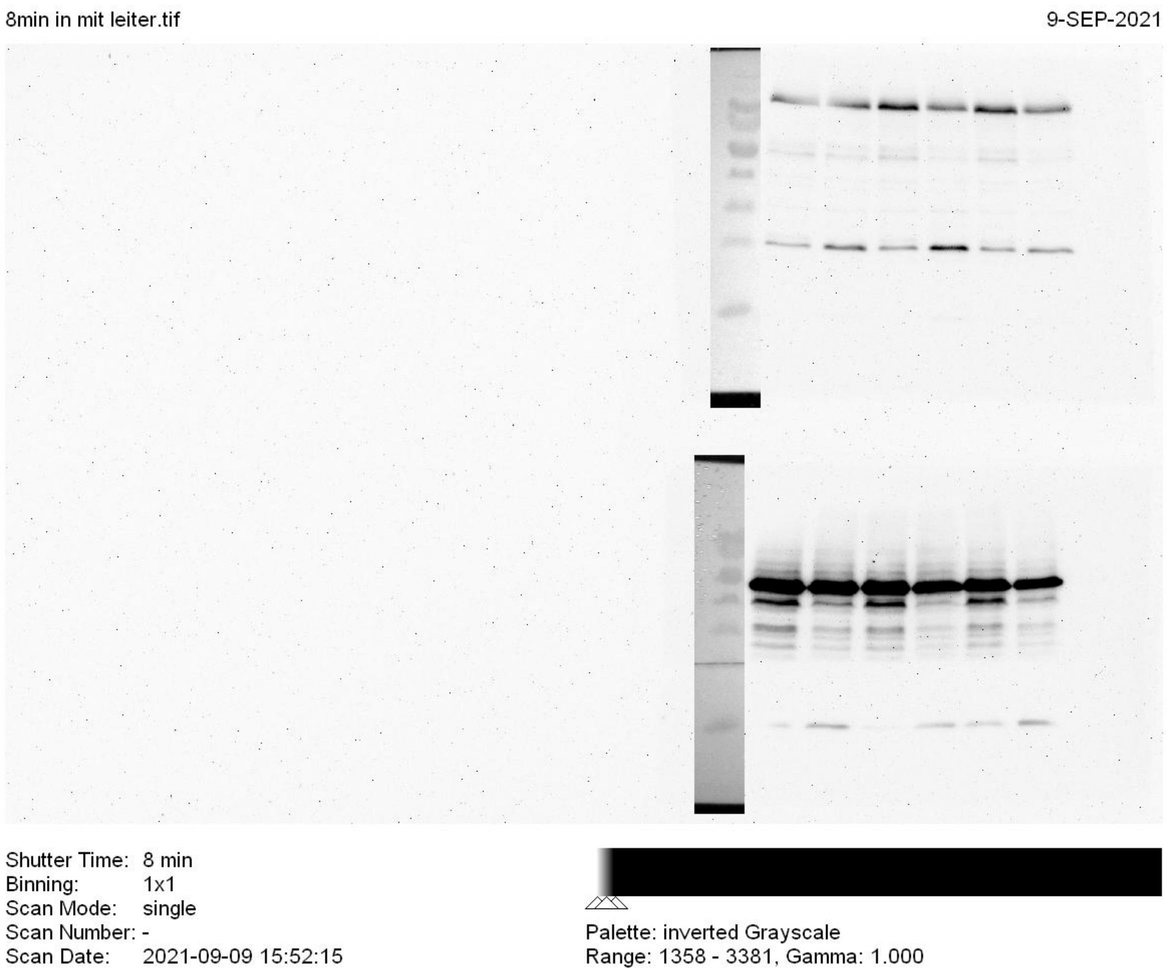


**Figure S20.** Uncropped western blot, MONO-MAC-6, blot at top shows PARP, blot below illustrate γ-H2AX, 48 h incubation.

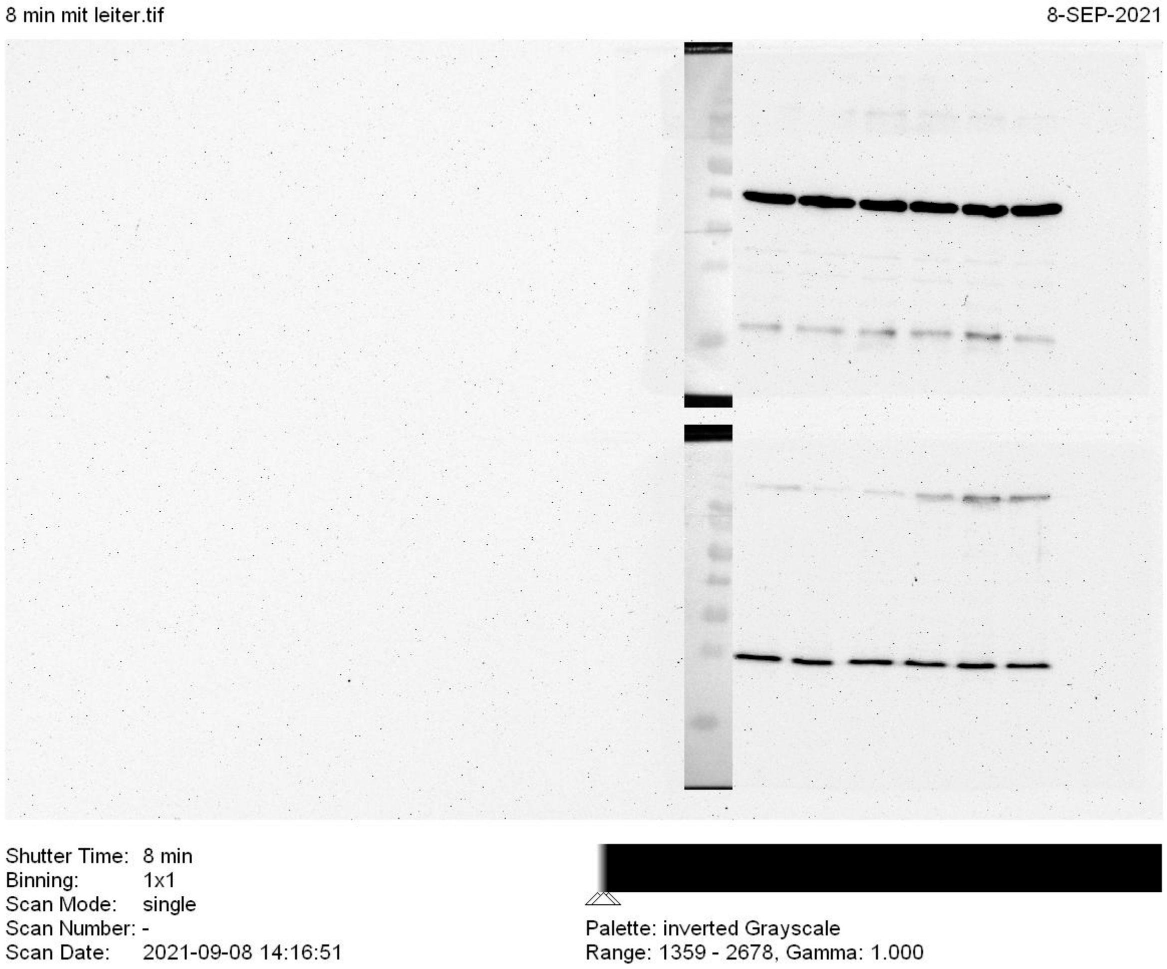


**Figure S21.** Uncropped western blot, MONO-MAC-6, blot at top shows APAF-1 and Survivin, blot below displays HDAC 4 and Bcl-2, 48 h incubation.

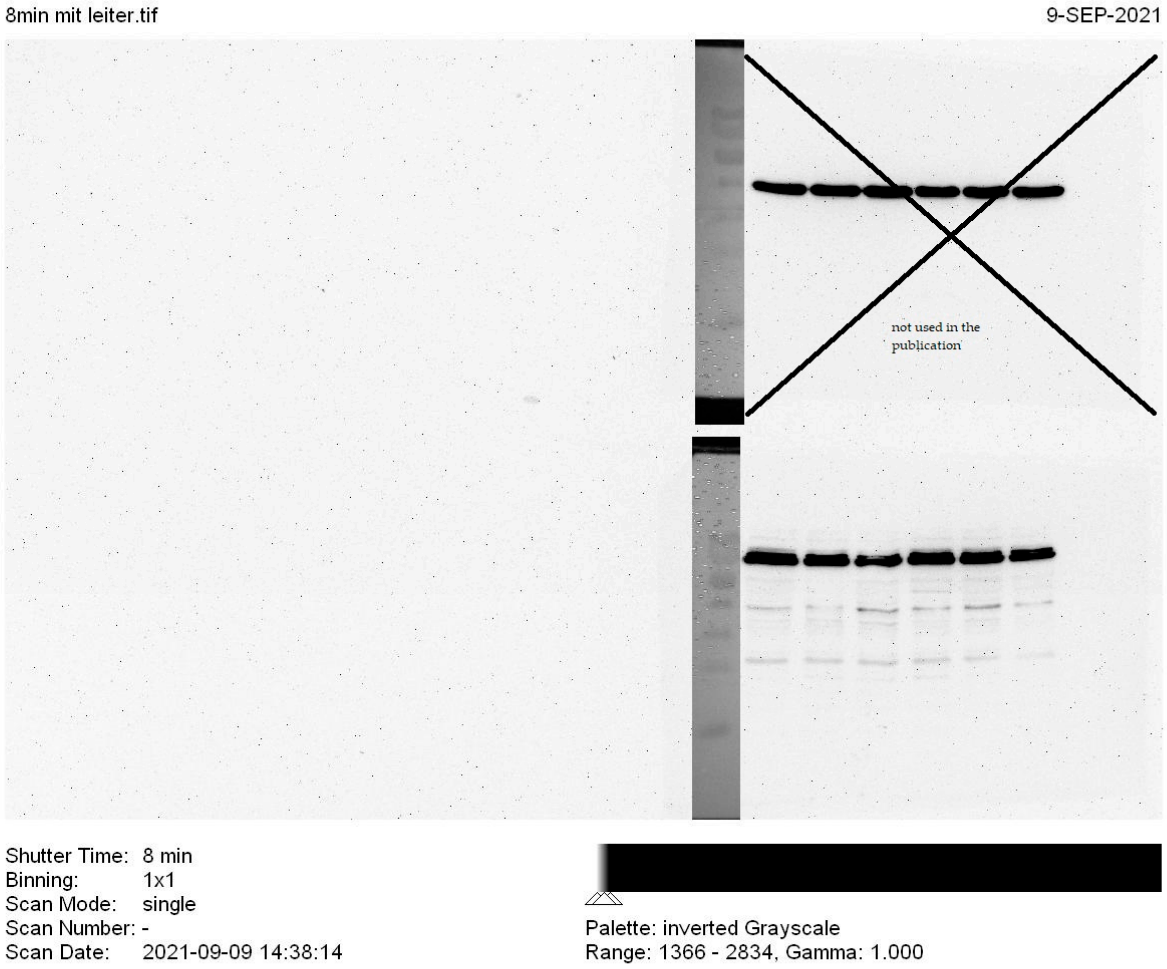


**Figure S22.** Uncropped western blot, MONO-MAC-6, blot below shows Bad, 48 h incubation.

**Caspase 3/7 fluorescent imaging pictures**

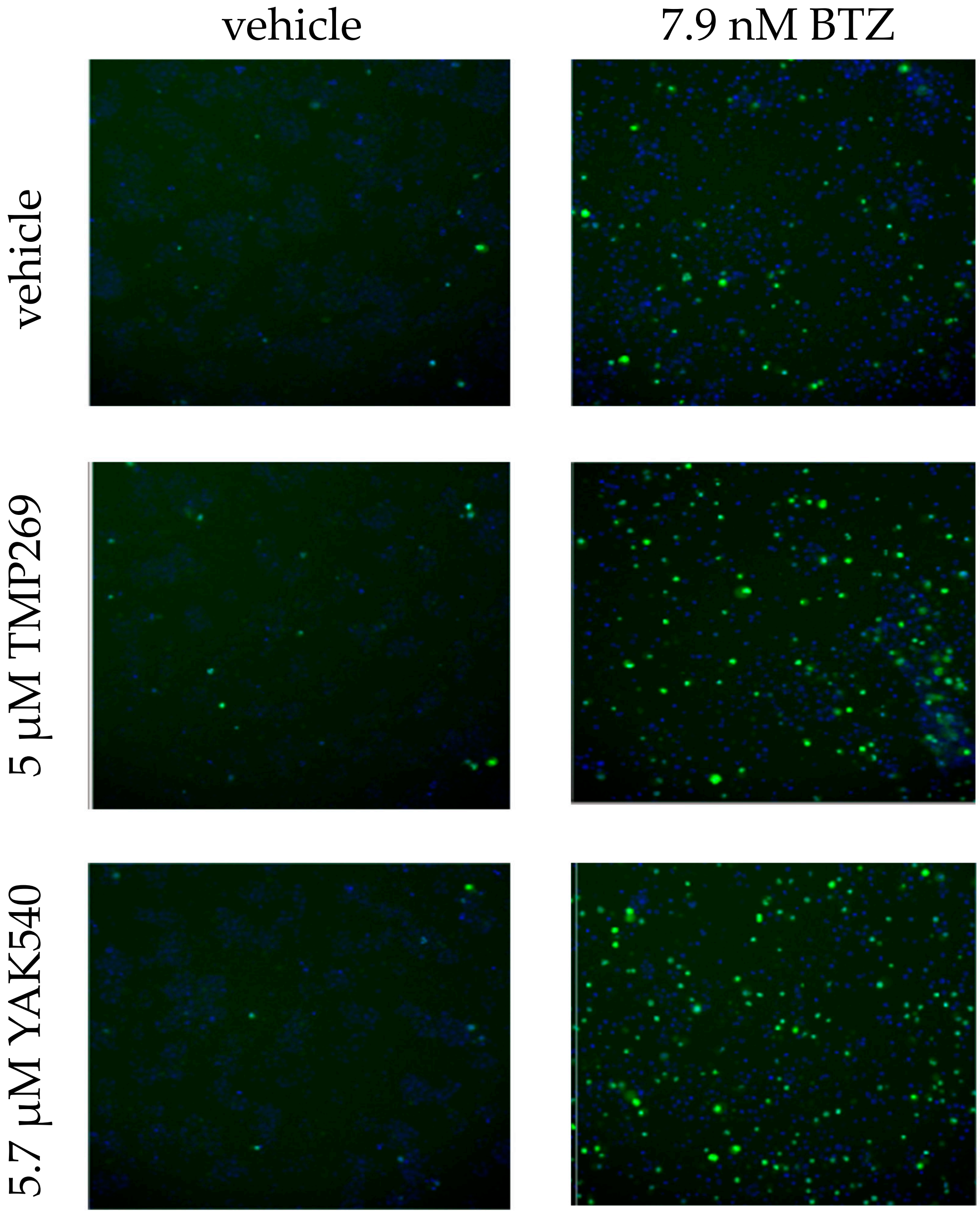


**Figure S23.** Representative fluorescent imaging pictures (Thermofisher Arrayscan XTI) are shown for vehicle control, treatment with 7.9 nM BTZ, 5 μM TMP269, 5.7 μM YAK540, respectively, and combination treatments of BTZ with TMP269 or YAK540 in THP-1 cells. Nuclei were stained using Hoechst 33342 (blue), cells showing caspase 3/7 activation are shown in green.
